# Differential role of intravenous anesthetics in colorectal cancer progression: implications for clinical application

**DOI:** 10.18632/oncotarget.12800

**Published:** 2016-10-21

**Authors:** Fengliu Deng, Mingwen Ouyang, Xiaofei Wang, Xueqing Yao, Yeming Chen, Tao Tao, Xuegang Sun, Lijun Xu, Jing Tang, Liang Zhao

**Affiliations:** ^1^ Department of Pathology, Nanfang Hospital, Southern Medical University, Guangzhou, Guangdong, China; ^2^ Department of Pathology, School of Basic Medical Sciences, Southern Medical University, Guangzhou, Guangdong, China; ^3^ Department of anesthesia, Fifth Affiliated Hospital of Southern Medical University, Guangzhou, Guangdong, China; ^4^ Department of Anesthesia, Nanfang Hospital, Southern Medical University, Guangzhou, Guangdong, China; ^5^ Department of General Surgery, Guangdong General Hospital, Guangdong Academy of Medical Science, Guangzhou, Guangdong, China; ^6^ Department of Anesthesia, Huarui Hospital, Southern Medical University, Guangzhou, Guangdong, China; ^7^ The Key Laboratory of Molecular Biology, State Administration of Traditional Chinese Medicine, School of Traditional Chinese Medicine, Southern Medical University, Guangzhou, Guangdong, China

**Keywords:** epithelial-mesenchymal transition, colorectal cancer, dexmedetomidine, propofol, etomidate

## Abstract

Anesthetics are unavoidable to colorectal cancer (CRC) patients who underwent surgical treatment. Thus, the molecular mechanisms underlying the role of the intravenous anesthetics in CRC metastasis are still unclear. In this study, the effects of intravenous anesthetics, such as propofol, etomidate and dexmedetomidine, on cell migration were determined. The migration of CRC cells was inhibited by propofol *in vitro*, but not *in vivo*. Etomidate, however, promoted the migration of CRC cells both *in vitro* and *in vivo*. Epithelial-mesenchymal transition (EMT) mediated the promotive effect of propofol and etomidate on the migration of CRC cells through PI3K/AKT signaling pathway. Dexmedetomidine alone or in combination with propofol or etomidate had minor effect on the migration of CRC cells. These findings indicate that propofol inhibites CRC cell migration *in vitro*. Etomidate playes a role for prompting CRC metastasis progression by activating (PI3K)/AKT signaling and inducing EMT. It provides an important hint for the clinical application of these anesthetics.

## INTRODUCTION

Cancer is a major cause of death in human beings worldwide. Colorectal cancer (CRC) is the third most common type of cancer making up about 10% of all cases. [1] Thus, surgery still remains the most effective and mainstay treatment option for CRC. [2] However, in the first five years after surgery, 20 to 30% of patients develop recurrent disease with greater than 90% of recurrences occurring ultimately. [3] So it is critically important to understand and define the factors during the perioperative period, which have effects on the longer-term outcome of patients after surgery, especially in CRC recurrence and progression. Since CRC patients must endure anesthesia for surgery, anesthetics are unavoidable and the subject of much speculation in recent years.

**Figure 1:**
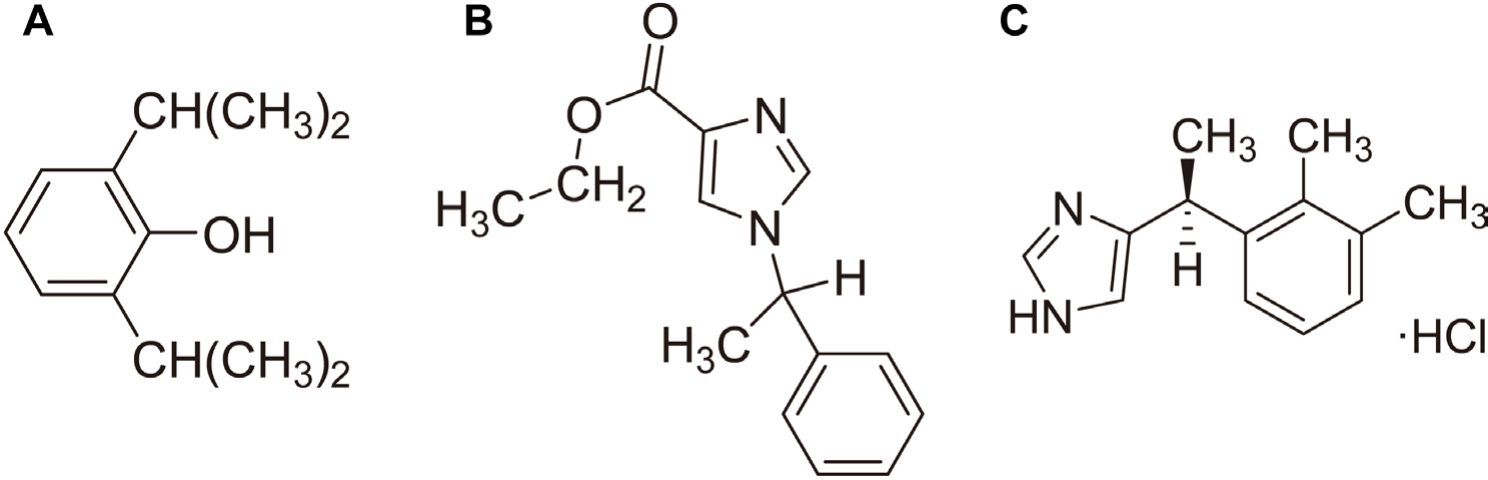
Chemical structure of propofol (A), etomidate (B) and dexmedetomidine (C)

Due to the characteristics of regaining full consciousness rapidly without accumulation during continuous infusion and fast onset of action, both propofol (2,6-diisopropylphenol, Figure 1A) and etomidate (R-(2-ethyl 1-(phenylethyl)-1H-imidazole-5-carboxylate), Figure 1B) are widely used in the induction and maintenance of anesthesia for patients undergoing CRC resection surgery. [4, 5] Propofol also has the anti-inflammatory and anti-oxidant effects. [6, 7] It has been proven that propofol can inhibit the adhesion of neutrophil to vascular endothelial cells, scavenge oxygen free radicals, alleviate oxidative damage, and decrease the release of inflammatory cytokines such as TNF-α, IL-6 and IL-1β. [8–10] Clinically used concentrations of propofol are reported to promote apoptosis and inhibit the invasion of human cancer cells. [11, 12] In cervical cancer, propofol decreases cell viability and induces cell apoptosis through down-regulating HOTAIR mediated mTOR/p70S6K pathway. [13] Conversely, some other researchers found propofol can activate Nrf2 leading to the proliferation and invasion of gallbladder cancer cells. [14] However, it remains unclear that propofol play a role in CRC metastasis. Etomidate is characteristic of short-acting property, as well as low cardiovascular risk profile. Compared with other induction agents, etomidate is less likely to cause an obvious drop in blood pressure. [15] Although both propofol and etomidate are common and widely used intravenous anesthetics, the study focusing on the effect of etomidate on cancer cells is rare. Dexmedetomidine (4-[(1S)-1-(2,3-Dimethylphenyl)ethyl]-1H-imidazolehydrochloride, Figure 1C) is an agonist of α2-adrenergic receptors and notable for its ability to provide sedation without risk of respiratory depression and can provide cooperative or semi-arousable sedation. [16] During surgery, dexmedetomidine is often used to reduce the dosage of general anesthetics such as propofol and etomidate. [17] The effect of dexmedetomidine on cancer cell migration needs to be further elucidated.

Since surgery is the best and most effective way to treat CRC patients, it is clinically important and meaningful to figure out how these anesthetics are affecting cancer cells. To solve these problems, colorectal cancer cell lines SW480 and HCT116 were used to determine the effect of propofol, etomidate and dexmedetomidine on these cancer metastasis.

## RESULTS

### The effects of propofol, etomidate and dexmedetomidine on the migration ability of CRC cells

**Figure 2:**
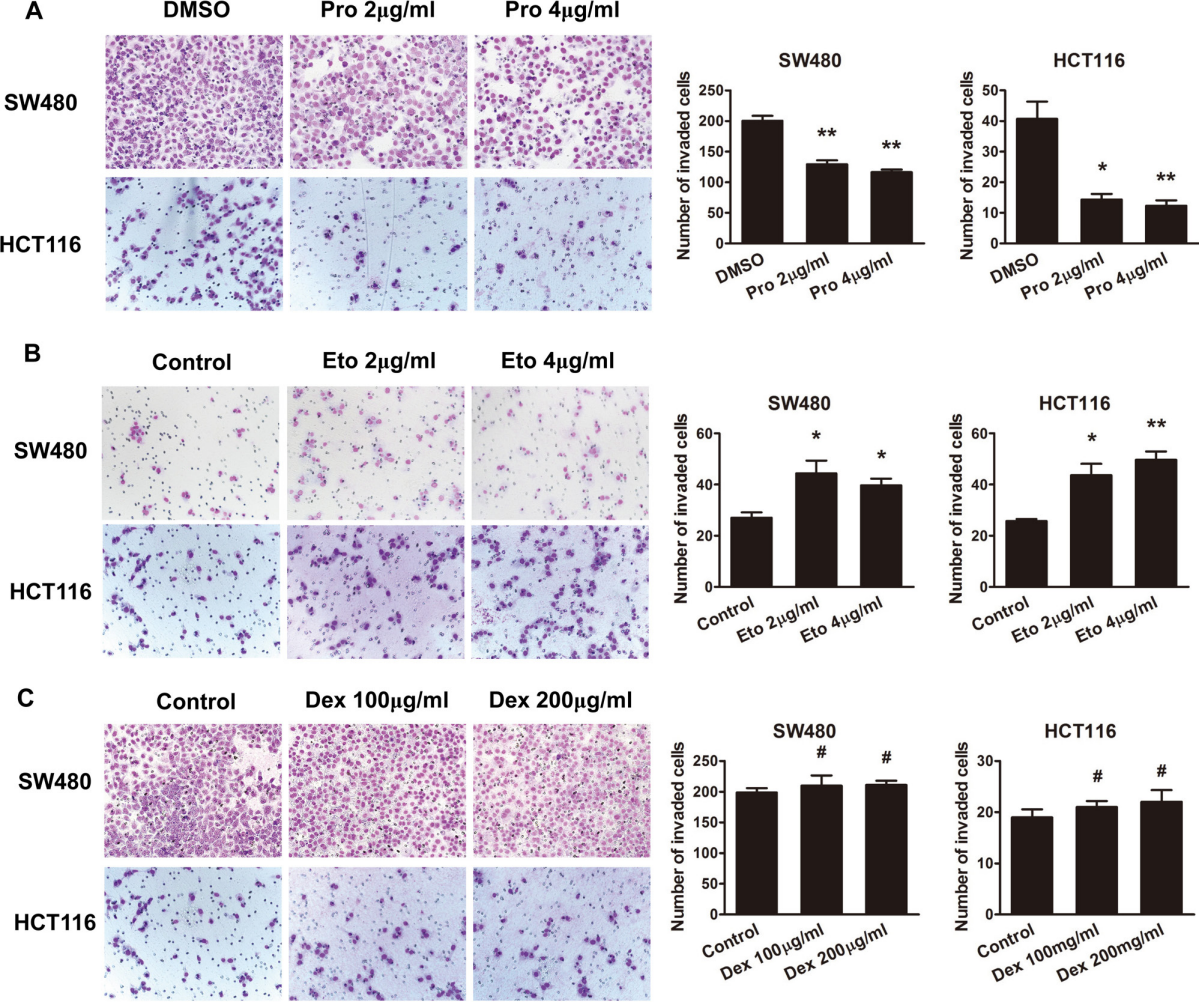
The effects of propofol, etomidate and dexmedetomidine on the migration ability of CRC cells (**A**) Representative figures and data of transwell assay for SW480 and HCT116 cells treated with 2 and 4 μg/ml of propofol for 24 h. (**B**) Representative figures and data of transwell assay for SW480 and HCT116 cells treated with 2 and 4 μg/ml of etomidate for 24 h. (**C**) Representative figures and data of transwell assay for SW480 and HCT116 cells treated with 100 and 200 μg/ml of dexmedetomidine for 24 h. Each bar represented the mean ± SD. The results were reproduced in three independent experiments. The asterisk (*) indicates *P* < 0.05. The asterisk (**) indicates *P* < 0.01.

To determine whether intravenous anesthetics regulate the capacity of cell migration, we performed transwell assay to evaluate aggressive capacity of CRC cells. The results showed that the ability of cell migration was suppressed by propofol in a dose-independent manner in SW480 and HCT116 cells (Figure 2A). The opposing results were observed in SW480 and HCT116 cells exposed to etomidate (Figure 2B). However, dexmedetomidine has no effect on CRC cell migration. (Figure 2C).

**Figure 3:**
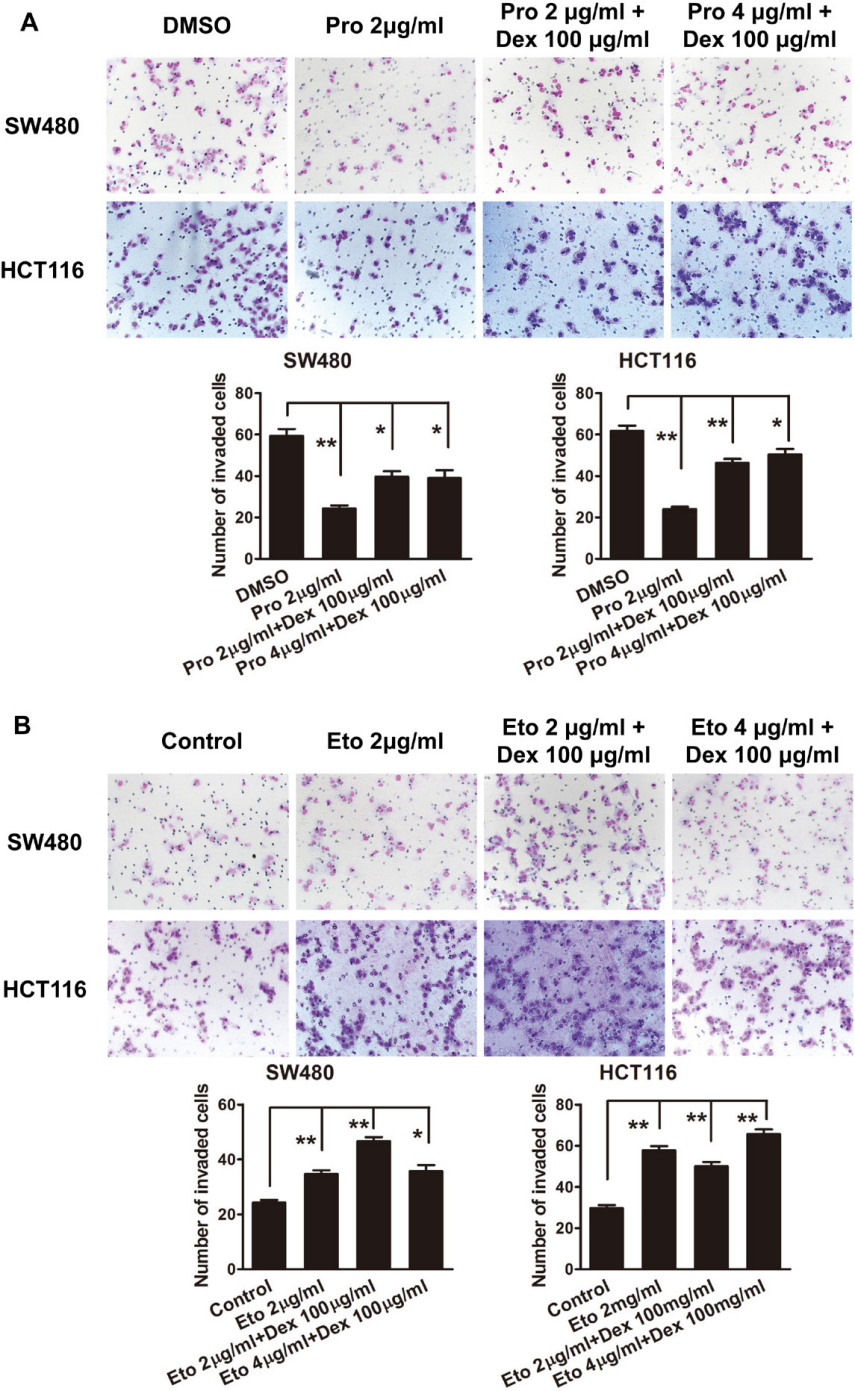
Propofol decreases and etomidate increases the migration ability of CRC cells (**A**) Representative figures and data of transwell assay for SW480 and HCT116 cells treated with 100 μg/ml of dexmedetomidine in combination with 2 or 4 μg/ml of propofol for 24 h. (**B**) Representative figures and data of transwell assay for SW480 and HCT116 cells treated with 100 μg/ml of dexmedetomidine in combined with 2 or 4 μg/ml of etomidate for 24 h. Each bar represented the mean ± SD. The results were reproduced in three independent experiments. The asterisk (*) indicates *P* < 0.05. The asterisk (**) indicates *P* < 0.01.

To evaluate the effects of dexmedetomidine on propofol or etomidate-mediated cell aggressive behaviors, we treated CRC cells with a combination of dexmedetomidine with propofol or etomidate. As shown in Figure 2, dexmedetomidine has no effect on propofol-suppressed cell migrationin cell migration (Figure 3A). Similarly, dexmedetomidine has no effect on etomidate-promoted the aggressive phenotype of CRC cells. (Figure 3B).

### Propofol decreases and etomidate increases AKT activation and epithelial-mesenchymal transition (EMT)

**Figure 4:**
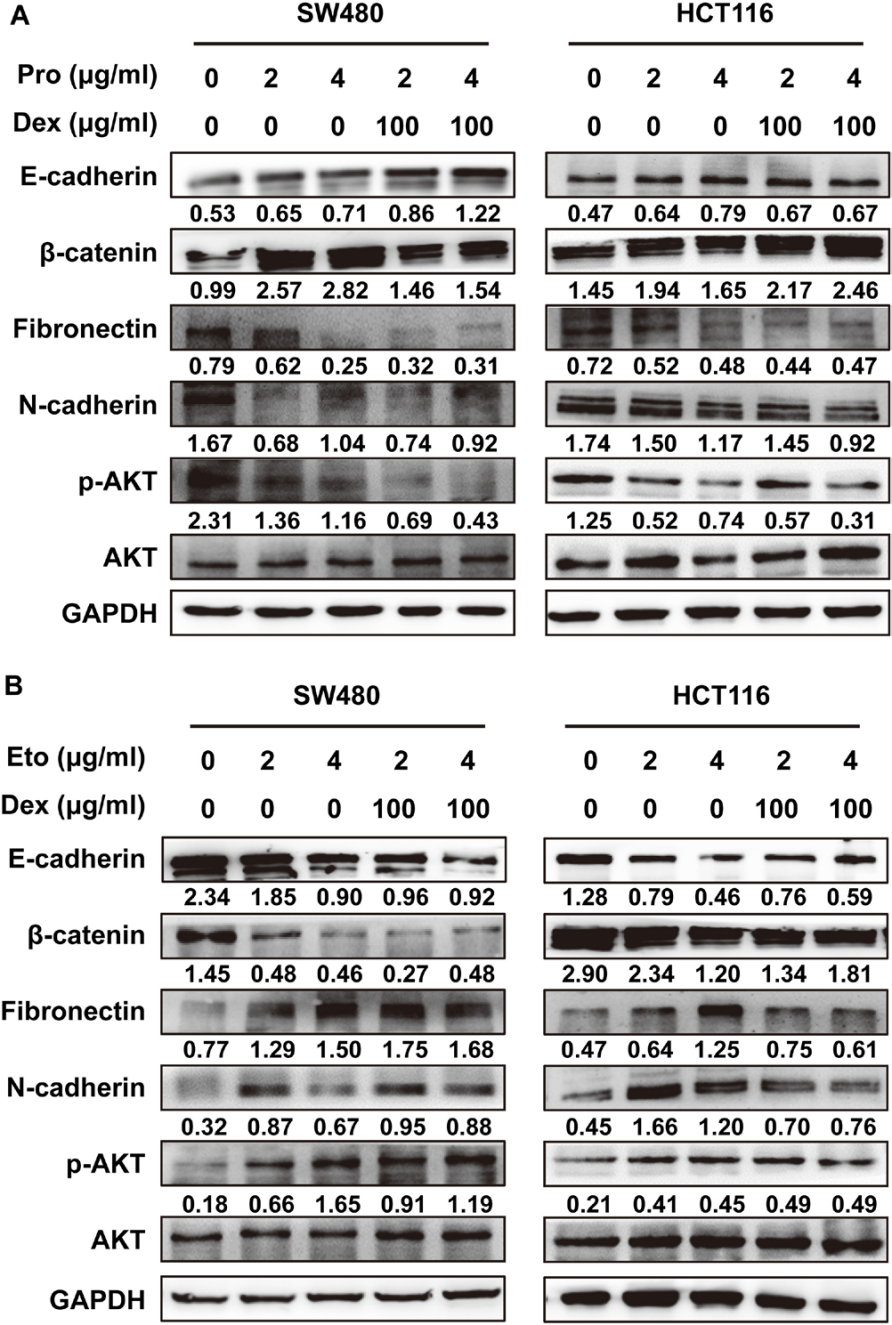
Propofol decreases and etomidate increases AKT activation and epithelial-mesenchymal transition (EMT) (**A**) Western blotting analysis of EMT markers and phosphorylated AKT in SW480 and HCT116 cells treated with 100 μg/ml of dexmedetomidine in combined with 2 or 4 μg/ml of propofol for 24 h. (**B**) Western blotting analysis of EMT markers and phosphorylated AKT in SW480 and HCT116 cells treated with 100 μg/ml of dexmedetomidine in combined with 2 or 4 μg/ml of etomidate for 24 h. Representative figures were shown. The results were reproduced in 3 independent experiments.

To uncover the mechanism underlying CRC cells phenotype induced by propofol and etomidate, we performed western blot analysis to detect the phosphorylation level of AKT and the expression of EMT markers. As shown in Figure 4A, propofol alone or in combination with dexmedetomidine inhibited EMT-like changes, which indicated by the increased expression of epithelial markers (E-cadherin and β-catenin) and the decreased expression of mesenchymal marker (fibronectin and N-cadherin) in SW480 and HCT116 cells. Meanwhile, the phosphorylation level of AKT was decreased in cells to propofol. In contrast, we found that etomidate can assume the EMT process and increase phosphorylation of AKT in SW480 and HCT116 cells (Figure 4B).

### AKT activation is responsible for the promotive effect of etomidate on cell migration

**Figure 5:**
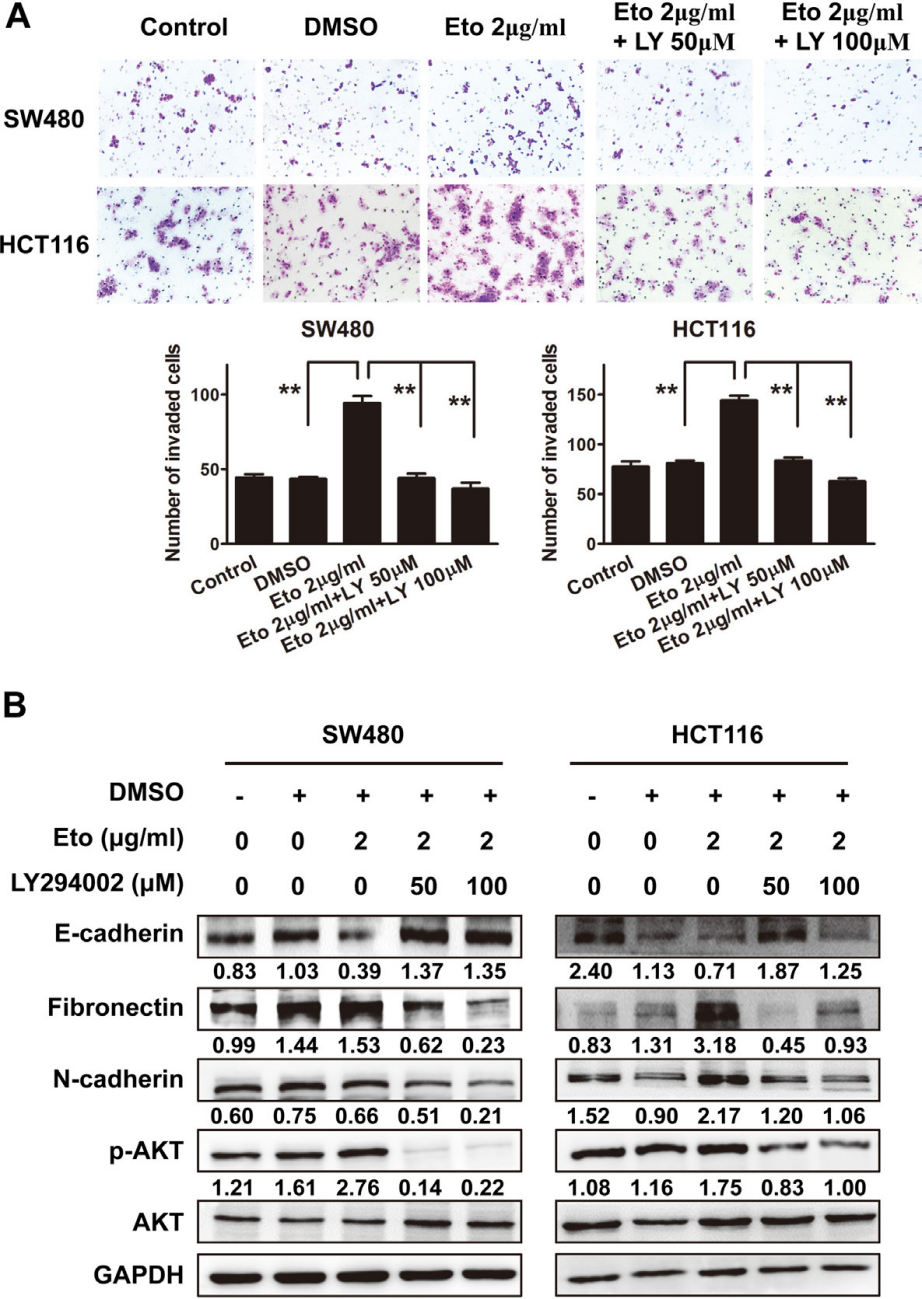
AKT activation is responsible for the promotive effect of etomidate on cell migration (**A**) Representative figures and data of transwell assay for indicated cells. Each bar represented the mean ± SD. The results were reproduced in three independent experiments. The asterisk (*) indicates *P* < 0.05. The asterisk (**) indicates *P* < 0.01. (**B**) Western blotting analysis of EMT markers and phosphorylated AKT in indicated cells. Representative figures were shown. The results were reproduced in 3 independent experiments.

To address the pivotal role of PI3K/AKT pathway in etomidate-mediated aggressive phenotype, we blocked the pathway to observe the ability of cell migration and the occurrence of EMT in CRC cells. Treatment of PI3K inhibitor LY294002 counteracted aggressive phenotype induced by etomidate (Figure 5A). Western blot analysis showed that LY294002 markedly suppressed phosphorylation of AKT and reversed EMT induced by etomidate in SW480 and HCT116 cells (Figure 5B).

### Etomidate, but not propofol, promotes CRC progression *in vivo*

**Figure 6:**
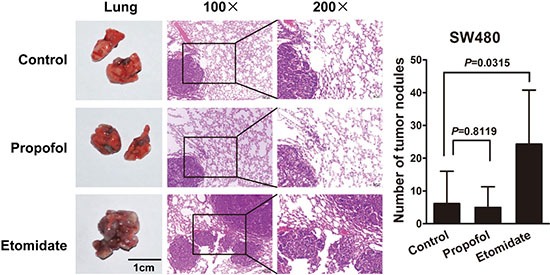
Etomidate, but not propofol, promotes CRC progression *in vivo* Mice were anesthesia with an intraperitoneal injection of propofol 20 mg/kg or etomidate 30 mg/kg. Tumor cells were injected into nude mice through the tail vein to evaluate the lung homing potential of cells. The number of metastatic lung nodules in individual mice was counted under the microscope. The magnification areas indicated metastatic nodes in the lung.

To observe the effect of propofol and etomidate on the potential of homing capacity, nude mice were injected with cancer cells through tail vein to observe lung nodules formation. Compared to control group, more and larger tumor nodules were found in the lung of mice with etomidate-treated groups. However, there was no significant difference in cancer metastasisbetween control and propofol-treated groups (Figure 6).

## DISCUSSION

Anesthetics are unavoidable for colorectal cancer patients who received surgical treatment. But whether these anesthetics would affect the long term outcome and especially the recurrence of CRC patients is always ignored in clinics. Interestingly, recently accumulating clinical evidence indicated that both anesthetics and the method of anesthesia affect the outcomes in patients undergoing surgery for a variety of cancers. [18–20] So it is meaningful for us to observe the effect of intravenous anesthetics, such as propofol, etomidate and dexmedetomidine, on the metastasis of colorectal cancer cells both *in vitro* and *in vivo*. Consistent with the previous researches [13, 14], we found that propofol suppresses cell migration of SW480 and HCT116 CRC cells in a dose-independent manner. Surprisingly, etomidate promotes the migration of CRC cells. However, the molecular mechanisms underlying the role of intravenous anesthetics in CRC metastasis still remains unclear.

Recent studies have illustrated that epithelial-mesenchymal transition (EMT) played a critical and initiating role in cancer progression and metastasis. EMT is a biological process which permits a polarized epithelial cell to undergo multiple biochemical changes, thereby enabling it to assume a mesenchymal cell phenotype. [21] Cancer cells undergoing EMT acquire aggressive properties and enter the surrounding stroma. [22] EMT is also a common molecular mechanism in CRC metastasis. [23, 24] Taking this into consideration, we hypothesized that EMT mediates anesthetic agents-altered CRC metastasis. In the study, we first reported that etomidate could induce EMT initiation, whereas propofol reversed EMT process, in the progression of CRC

Several studies demonstrated the involvement of intravenous anesthetics in regulating key signaling pathways. Propofol inhibited LPS-induced AKT activation and phosphorylation (Ser473) partly by reducing reactive oxygen species generation, which was crucial for LPS-induced inflammatory responses in macrophages. [10] Meanwhile, pretreatment with low dose of etomidate increased extracellular signal-regulated kinase1/2 activation, but suppressed AKT activation. [25] Furthermore, two distinct binding cavities were identified within the β2 subunit of the transmembrane domain in a molecular model of the GABAA protein, which are hypothesized to be distinct binding sites for etomidate and propofol. [26] Functional evidence supported that these intravenous anesthetics either modulates through a different set of subunit interfaces or through the same set of subunit interfaces to a different degree. [27] The role and molecular mechanism of etomidate and propofol in cancer, however, is still unknown. In this study, etomidate activates and propofol suppresses PI3K/AKT pathway, which was recognized as a pivotal link of cancer progression. Novel receptors in cancer cells were needed to be further identified.

Accumulating genetic and cancer biology evidence demonstrated that activation of the PI3K/AKT axis is emerging as a central feature of EMT, despite its definite effects on cancer cell growth and aggressiveness. [28] Galectin-1 (Gal1), a β-galactoside-binding protein abundantly expressed in tumor microenvironments, induced E-cadherin downregulation and triggered EMT through a PI3K/Akt-dependent mechanism in human hepatocellular carcinoma cells. [29] In gastric cancer, the activated Cdc42-associated kinase 1 (ACK1) phosphorylated AKT and activated the AKT pathway to induce EMT and promote cell migration and invasion. [30] Administration of mice with the PI3K inhibitor LY294002 resulted in the reversion of EMT, with reduced pulmonary metastasis of nasopharyngeal carcinoma cells. [31] Our data showed that LY294002 suppresses etomidate-mediated EMT and migration of CRC cells, suggesting its clinical significance of inhibition of AKT signaling in combination with intravenous anesthetics during the perioperative period.

Animal experiments were performed to simulate clinical application of three intravenous anesthetics in CRC patients who undergo resection surgery. To amplify the effects of anesthetics on tumor cells *in vivo*, mice were treated with anesthetics once a week within two months. Since the superficial veins of mouse is very slim and small and both peopofol and etomidate are fat emulsion solvent which is difficult to inject through small superficial veins and easier to exude out of the veins, so intraperitoneal injection as our mode of administration. In contrast to the findings *in vitro*, propofol seemed to slightly suppress the number and size of metastatic nodules in the lung of mice. No change in lung homing capacity of tumor cells was found after treatment with propofol. *In vivo*, the migration of tumor cells can be affected by many factors such as immune cells, cytokines and tumor microenvironment besides propofol. [32–34] So this may be the reason why the difference occurred between *in vitro* and *in vivo* experiments concerning about the effect of propofol on tumor cells migration, which needs to be further studied. Our present results support that propofol is a relatively safe anesthetic for cancer patients especially the CRC patients. On the other hand, our study first revealed that etomidate promotes the migration of CRC cells both *in vitro* and *in vivo*. These results strongly suggest that etomidate should be carefully used in CRC patients during surgery, although it has the advantage of a relatively safer cardiovascular risk profile. Finally, dexmedetomidine has no effect on CRC cells whenever used alone or in combination with other intravenous anesthetics such as propofol or etomidate.

In general, we investigated the effects of three most commonly used intravenous anesthetics, propofol, etomidate and dexmedetomidine, on the migration of CRC cells both *in vitro* and *in vivo*. Interestingly, we found propofol obviously inhibits the migration of CRC cells *in vitro* but not *in vivo*. Etomidate, on the contrary, promotes the aggressive phenotype of CRC cells both *in vitro* and *in vivo*. EMT mediates the suppressive effect of propofol and the promotive effect of etomidate on CRC cell migration through PI3K/AKT pathway. Dexmedetomidine, however, showed minor effect on the aggressive phenotype of CRC cells whenever used alone or in combination with propofol and etomidate. These results provide important information that may be useful regarding the clinical application of these anesthetics. During the process of surgery, propofol and dexmedetomidine are relatively safe for CRC patients. Etomidate, however, should be carefully used in this kind of patients because of its role for prompting CRC progression.

## MATERIALS AND METHODS

### Cell culture

The colorectal cancer cell lines SW480 and HCT116 were obtained from the Chinese Academy of Sciences (Shanghai, China). All cell lines were cultured in monolayer in 75-cm^2^ tissue culture flasks maintained at 37^°^C in humidified air balanced with 5% CO_2_ in RPMI 1640 medium and10% heat-inactivated fetal calf serum. Culture medium was replaced every 48 h.

### Propofol, etomidate and dexmedetomidine treatment

Propofol (AstraZeneca, Sweden/UK) was dissolved in DMSO (Sigma-Aldrich, Dorset, UK). Etomidate (Enhua Medicine, Jiangsu, China) and dexmedetomidine (HengruiCo., Jiangsu, China) were dissolved in serum-free media. Before treatment, CRC cells were cultured at the density of 1 × 10^6^ per ml on 30-mm^2^ Petri dishes, as described above. Drugs-supplemented medium was then added to the cell cultures for 2 h. Cells were then washed with phosphate-buffered saline (PBS; GIBCO, Invitrogen) and replaced with normal cell culture medium. Cells were harvested at 24 h for the following treatment.

### Inhibitor treatment

PI3k inhibitor LY294002 (Cell Signal Technology, Danvers, MA) was diluted in serum-free media. 10 mmol/L LY294002 was added in the cultured cells. Cells were harvested at 24 h for the following treatment. Two hours prior to harvesting, LY294002 was added again as before.

### Transwell assay

Invasion of cells was evaluated with the Cell Invasion Assay Kit (BD Biosciences) following the manufacturer's instructions. Briefly, after 24 h of treatment with anesthesia, 1 × 10^5^ cells in 300 μL serum-free medium were added to the upper chamber. 0.7 mL medium with 20% FBS was added to the lower chamber as a chemoattractant. Subsequently, cells were incubated at 37°C for 48–72 h. Furthermore, non-invading cells were removed with cotton swabs. Cells that migrated to the bottom of the membrane were fixed with pre-cold methanol and stained with 2% Giemsa solution. Finally, Stained cells were visualized under a microscope. To minimize the bias, at least three randomly selected fields with 100 × magnification were counted, and the average number was taken.

### Western blot

Total protein was harvested by RIPA lysis buffer with Protease Inhibitor Cocktail, and quantified using the BCA protein assay kit (Pierce). This protein was separated by SDS-PAGE gel and transferred onto PVDF membrane (Millipore). The membranes were incubated with rabbit antibodies to β-actin, fibronectin (1:500; Santa Cruz, California, USA) and rabbit antibodies to β-catenin, E-catenin, N-cadherin, p-AKT(Ser473), AKT (1:1000; CST, Danvers, MA) overnight, and followed by HRP-conjugated secondary antibody (1:10000; CST, Danvers, MA), respectively. The signal was detected using enhanced chemiluminescence detection system (Pierce, Rockford, IL) as described by the manufacturer.

### Animal studies

All procedures involving animal experiments were approved by The Southern University Animal Care and Use Committee and in conformity with NIH guidelines. Four-six weeks-old female nu/nu mice were used. Animal subjects were divided into three groups of six mice per group and housed with free access to food and water. To verify the *in vivo* effect of propofol and etomidate on colorectal cancer, mice were anesthetized with an intraperitoneal injection of propofol 20 mg/kg or etomidate 30 mg/kg. And then mice were injected intravenouslywith SW480 cells 1 × 10^7^ dissolved in phosphate-buffered saline (PBS). Control (NC) was free of drugs. The experimental animals were given drugs once a week. Two months later, mice were sacrificed and tumors were removed. The number and size of tumors were calculated.

### Statistical analysis

All data are presented as means ± standard deviation from three independent experiments. SPSS19.0 software was used to carry out statistical analysis. The differences between groups were investigated by student's *t*-test with only two groups or one-way analysis of variance (ANOVA) when more than two groups were compared. *P* < 0.05 was considered statistically significant.
